# Mesenchymal stromal cells ameliorate oxidative stress-induced islet endothelium apoptosis and functional impairment via Wnt4-β-catenin signaling

**DOI:** 10.1186/s13287-017-0640-0

**Published:** 2017-08-14

**Authors:** Lingshu Wang, Li Qing, He Liu, Na Liu, Jingting Qiao, Chen Cui, Tianyi He, Ruxing Zhao, Fuqiang Liu, Fei Yan, Chuan Wang, Kai Liang, Xinghong Guo, Ying H. Shen, Xinguo Hou, Li Chen

**Affiliations:** 1 Department of Endocrinology, Institute of Endocrinology and Metabolism, Qilu Hospital of Shandong University, Jinan, 250012 Shandong China; 2 College of Public Health, Shandong University, Jinan, Shandong 250012 China; 3 Division of Cardiothoracic Surgery, Michael E. DeBakey Department of Surgery, Baylor College of Medicine, Houston, TX USA; 4 Texas Heart Institute, Houston, TX USA

**Keywords:** Islet endothelium, Mesenchymal stromal cell, Wnt

## Abstract

**Background:**

Islet dysfunction and destruction are the common cause for both type 1 and type 2 diabetes mellitus (T2DM). The islets of Langerhans are highly vascularized miniorgans, and preserving the structural integrity and full function of the microvascular endothelium is vital for protecting the islets from the infiltration of immune cells and secondary inflammatory attack. Mesenchymal stromal cell (MSC)-based therapies have been proven to promote angiogenesis of the islets; however, the underlying mechanism for the protective role of MSCs in the islet endothelium is still vague.

**Methods:**

In this study, we used MS-1, a murine islet microvascular endothelium cell line, and an MSC-MS1 transwell culturing system to investigate the protective mechanism of rat bone marrow-derived MSCs under oxidative stress in vitro. Cell apoptosis was detected by TUNEL staining, annexin V/PI flow cytometry analysis, and cleaved caspase 3 western blotting analysis. Endothelial cell activation was determined by expression of intercellular cell adhesion molecule (ICAM) and vascular cell adhesion molecule (VCAM), as well as eNOS phosphorylation/activation. The changes of VCAM-1, eNOS, and the β-catenin expression were also tested in the isolated islets of T2DM rats infused with MSCs.

**Results:**

We observed that treating MS-1 cells with H_2_O_2_ triggered significant apoptosis, induction of VCAM expression, and reduction of eNOS phosphorylation. Importantly, coculturing MS-1 cells with MSCs prevented oxidative stress-induced apoptosis, eNOS inhibition, and VCAM elevation in MS-1 cells. Similar changes in VCAM-1 and eNOS phosphorylation could also be observed in the islets isolated from T2DM rats infused with MSCs. Moreover, MSCs cocultured with MS-1 in vitro or their administration in vivo could both result in an increase of β-catenin, which suggested activation of the β-catenin-dependent Wnt signaling pathway. In MS-1 cells, activation of the β-catenin-dependent Wnt signaling pathway partially mediated the protective effects of MSCs against H_2_O_2_-induced apoptosis and eNOS inhibition. Furthermore, MSCs produced a significant amount of Wnt4 and Wnt5a. Although both Wnt4 and Wnt5a participated in the interaction between MSCs and MS-1 cells, Wnt4 exhibited a protective role while Wnt5a seemed to show a destructive role in MS-1 cells.

**Conclusions:**

Our observations provide evidence that the orchestration of the MSC-secreted Wnts could promote the survival and improve the endothelial function of the injured islet endothelium via activating the β-catenin-dependent Wnt signaling in target endothelial cells. This finding might inspire further in-vivo studies.

## Background

Type 2 diabetes mellitus (T2DM) has become a global health issue due to its increasing morbidity and aggravating financial burden. According to ADA reports, the prevalence of T2DM in the USA has reached a plateau at approximately 12% [1]; however, the prevalence of diabetes and prediabetes is still increasing in China and has reached up to 11.6% and 50.1%, respectively, in the adult population of China [2]. With the development of T2DM, insulin resistance is present throughout the disease process; islet function exhibits a compensatory elevation in the early stage and undergoes a constant decline afterward. Thus, preserving islet function is the key in managing glucose and preventing T2DM progression.

The islets are highly vascularized miniorgans, with their combined 1–2% of the pancreatic volume receiving 10–20% of the total pancreatic blood flow [3]. Because of this unique structure, the islets are able to respond rapidly to glucose and hormone fluctuations but are also vulnerable to unfavorable stimuli such as oxidative stress. Once the microvascular integrity of the islets is impaired, together with the disturbance of the NO-mediated endothelium vasodilation [4] and the upregulation of adhesion molecules [5], it becomes easier for the inflammatory cells to adhere and migrate into the islet and cause further islet destruction [6]. Considering the importance of islet microvascular endothelium, preserving its integrity and proper function might be a novel target in islet protection.

Mesenchymal stromal cell (MSC)-based therapies have been proven effective in various clinical trials for both T1DM and T2DM [7, 8], as well as in diet-induced [9–11] or genetically modified experimental [12, 13] diabetic animal models. Systemic MSC infusion alone has been shown to alleviate hyperglycemia, improve islet function, and attenuate insulin resistance, while MSC and islet cotransplantation facilitates graft revascularization and promotes graft survival [14–17]. Relatively few studies have focused on changes in the existing islet microvascular endothelium after MSC transplantation [18], and the aforementioned studies analyzed the change in endothelial cell numbers but not the function of the islet vasculature, partially due to the difficulties of measuring the islet blood flow in vivo. Furthermore, the mechanism for the protective role of MSCs in islet endothelium is still vague. Considering the pro-angiogenesis nature of MSCs and their diverse secretome [19], it is reasonable to assume that MSCs could alter endothelial cell behavior and protect the islet endothelium from destructive stimuli by secreting various factors.

Therefore, in this study we used MS-1, a murine islet microvascular endothelium cell line, and rat primary bone marrow-derived MSCs to investigate the secreted factors and downstream pathways responsible for the protective effects of MSCs. To simulate a paracrine microenvironment, we built an MSC-MS1 transwell culturing system to investigate the protective mechanism of MSCs under oxidative stress, and the results might inspire further in-vivo studies.

## Methods

### Cell culture and treatments

Rat primary bone marrow MSCs (bmMSCs) were obtained by isolating the femurs of rats, flushing the marrow, and cultivating the eluent in Dulbecco’s modified Eagle’s medium (low glucose, 5.5 mmol/l (L-DMEM)) supplemented with 20% fetal bovine serum (FBS). After 24 h, the supernatant was discarded, and the culturing was continued until the cells reached 80% confluency. After the first passage, the MSCs were cultured in L-DMEM with 10% FBS. The third passage was used for conditioned medium (CM) gathering, differentiation induction, flow cytometry analysis for surface markers, and transwell culturing. The MSCs were cultured in the upper chamber of the transwell system (catalog no. 3414; Corning, USA).

The primary lung epithelial cells were obtained from newborn rats. The rats were sacrificed, and the lungs were separated, cut into small pieces, and digested in 0.25% trypsin. The cell suspension was cultivated in L-DMEM plus 10% FBS for 24 h, and the nonadherent cells were discarded.

MS-1 cells (MILE SVEN 1, ATCC Number: CRL-2279™) were cultured in H-DMEM supplemented with 5% FBS according to the supplier’s protocol (https://www.atcc.org/en/Global/Products/3/9/8/C/CRL-2279.aspx). Treatments were given after 24 h of serum starvation when the cells reached 60% confluency. For dose-dependent effects, MS-1 cells were treated with 0, 50, 100, 200, 400, and 800 μmol/L H_2_O_2_ for 24 or 48 h, and cell viability was detected using MTT. The optimal H_2_O_2_ concentration was that under which the cell viability dropped to 50–60% compared to cell viability of the control. The concentration of H_2_O_2_ used for the rest of the experiments was 200 μmol/L according to the results. The pharmacological inhibitor XAV-939 (catalog no. S1180; Selleckchem, USA), an inhibitor of β-catenin transcriptional activity, was incubated together with the other treatments to block the canonical Wnt signalling. The optimized concentration of XAV-939 was confirmed by treating MS-1 cells with 0, 2.5, 5, 10, 20, and 40 μmol/L XAV-939 for 24 h and extracting the proteins for western blotting analysis. The proper concentration was the minimal concentration that significantly reduced the amount of β-catenin while not increasing the proportion of cleaved caspase 3. Finally, 10 μmol/L of XAV-939 was used in the following experiments, and this concentration was consistent with that used in previous reports.

### Cell viability and apoptosis

Cell viability was detected using the MTT method. MS-1 cells were seeded in 96-well culture plates at 1 × 10^5^ cells/well. Four hours before analysis, 5 mg/ml MTT solution was added. For analysis, the supernatant was removed, and cells were solubilized in acid isopropyl alcohol. The absorption was measured by spectrophotometry at 570 nm with reference at 630 nm.

Cell apoptosis was detected using an In Situ Cell Death Detection kit (catalog no. 12156792910; Sigma-Aldrich, USA), FITC Annexin V Apoptosis Detection kit (catalog no. 556547; BD Pharmingen, USA), and western blotting analysis for the percentage of cleaved caspase 3 and 7.

In-situ cell death detection (TUNEL) was conducted following the manufacturer’s protocol. In brief, cells were planted on coverslips in six-well plates. After treatment, slides were washed three times with ice-cold PBS and fixed with 4% paraformaldehyde. The cells were incubated with 1% Triton X-100 for 5 min and then incubated at 37 °C with 50 μl/slide TUNEL reaction mixture in darkness for 60 min. After incubation, the slides were washed three times and stained with Hoechst33258 for 5 min. Apoptotic cells were counted in random fields by fluorescence microscopy; each experiment was performed in triplicate (×40 magnification, at least 10 fields per sample).

FITC annexin V apoptosis detection was performed using flow cytometry. Cells were planted on six-well plates. After treatment, cells were stained with annexin V for 20 min at room temperature in the dark, and propidium iodide (PI) was added 5 min before analysis.

### Immunocytochemistry

The nuclear transposition of β-catenin in MS-1 cells was detected by indirect immunofluorescence. The slides were prepared as already described. After incubation in 1% Triton X-100 for 5 min, the cells were incubated with 1:500 rabbit anti-rat β-catenin antibody (catalog no. ab32572; Abcam, USA) in 1% BSA in PBS overnight at 4 °C. The slides were incubated with goat anti-rabbit Alexa Fluor 488 (catalog no. 1515529; Life Technology, USA) diluted 1:500 in 1% BSA in PBS for 60 min and then DAPI for 10 min. The transposition of β-catenin was observed under laser scanning confocal microscopy.

### Real-time quantitative PCR

The total mRNA was extracted using an EZNA MicroElute Total RNA Kit (catalog no. R6831-01; Omega Bio-Tek, USA) under the manufacturer’s instructions and then reverse-transcribed using a Prime Script RT Reagent Kit (catalog no. RR047A; Takara, Japan). Primers were designed with Primer Premier 6.0 software and synthesized chemically by Sangon Biotech (Shanghai) Co., Ltd. The primers were as follows: Wnt2, sense 5′-CTCGGTGGAATCTGGCTCTG-3′ and antisense 5′-CACATTGTCACACATCACCCT-3′; Wnt3a, sense 5′-GTTTGCCGATGCCAGGGAGAA-3′ and antisense 5′-ACCACCAGCAGGTCTTCACTTC-3′; Wnt4, sense 5′-AGACGTGCGAGAAACTCAAAG-3′ and antisense 5′-GGAACTGGTATTGGCACTCCT-3′; Wnt5a, sense 5′-GCAGGTCAACAGCCGCTTCAACTC-3′ and antisense 5′-TCATAGCCACGCCCACAGCACAT-3′; and Wnt10b, sense 5′-GGACGCCAGGTGGTAACGGAAA-3′ and antisense 5′-GTCTCGCTCGCAGAAGTCAGGA-3′. Real-time PCR was conducted with the SYBR Green PCR kit (catalog no. RR820B; Takara), and quantification was achieved by normalization using β-actin as the control.

### Western blotting analysis

Whole-cell proteins were extracted by RIPA lysis buffer (catalog no. P0013B; Biotime, China). The plasma proteins were extracted by Tris-Triton (10 mM Tris, 100 mM NaCl, 1 mM EDTA, 1 mM EGTA, 1% Triton X-100, 10% glycerol, 0.1% SDS, and 0.5% deoxycholate), and the nuclear proteins were extracted by RIPA. Proteins were separated by 10% SDS-PAGE and transferred to nitrocellulose membranes. The membranes were blocked in 5% nonfat milk in TBS-T (50 mmol/l Tris, pH 7.5, 150 mmol/l NaCl, 0.05% Tween 20) for 1 h at room temperature and incubated in primary antibodies at 4 °C overnight. Bound primary antibodies were detected by horseradish peroxidase-conjugated secondary antibodies for 1 h at room temperature and visualized by enhanced chemiluminescence (Amersham Imager 600, GE, USA). Quantification of bands were performed using ImageJ software.

The primary antibodies were as follows: β-catenin, cyclin D1 (catalog no. ab134175; Abcam), Histone H3 (catalog no. 4499P; Cell Signaling Technology, USA), p-eNOS (catalog no. ab184154; Abcam), total-eNOS (catalog no. ab66127; Abcam), ICAM (catalog no. BA0541; Boster, China), VCAM (catalog no. XBA0406; Boster), Wnt4 (catalog no. sc376279; Santa Cruz, USA), Wnt5a (catalog no. sc365370; Santa Cruz), and β-actin (catalog no. BM0627; Boster), while cleaved/total caspase 3/7 were from an Apoptosis Antibody Sampler Kit (catalog no. 9915; Cell Signaling Technology, USA).

### Silencing RNA knockdown

Silencing RNA (siRNA) oligonucleotides were synthesized by Shanghai GenePharma Co., Ltd. The sequences of negative control (NC) siRNA were sense 5′-UCCUCCGAACGUGUCACGUTT-3′ and antisense 5′-ACGUGACACGUUCGGAGAATT-3′. The sequences for the Wnt4 siRNA were sense 5′-GCCAAGUCCAGACUUCUGUTT-3′ and antisense 5′-ACAGAAGUCUGGACUUGGCTT-3. The sequences for the Wnt5a siRNA were sense 5′-GAAGCCCAUUGGAAUAUUATT-3′ and antisense 5′-UAAUAUUCCAAUGGGCUUCTT-3′. The siRNAs for Wnt4 and Wnt5a could suppress the corresponding mRNA levels to less than 10% compared to that expressed in NC siRNAs, and the samples were also tested for other Wnt mRNAs to guarantee that the knockdown was specific and no off-target effects occurred (data not shown).

The siRNAs were transfected into rat MSCs using the Lipofectamine RNAiMAX Transfection Reagent (catalog no. 13778100; Invitrogen, USA) according to the manufacturer’s instructions. In brief, the MSCs were seeded in transwell chambers or in culture flasks until the cells reached 80–90% confluency. The medium was then removed and replaced with Opti-MEM I Reduced Serum Medium (catalog no. 31985070; Invitrogen), in which 1 × 10^6^ cells were subjected to 25 pmol siRNA mixed with 7.5 μl transfection reagent. After a 48-h incubation, the supernatant was discarded and was replaced with L-DMEM with 10% FBS. The efficiency of RNA knockdown was evaluated by qPCR and western blotting analysis. The cells in the transwell chamber were used for transwell culturing, and the supernatant was gathered as CM.

### Animals and bmMSC infusion

The T2DM rat model was established by a continuously high-fat diet (HFD) combined with a single dose of STZ (30 mg/kg, catalog no. S0130; Sigma-Aldrich) at the fourth week of HFD. Diabetes was identified as fasting glucose ≥ 16.7 mmol/L twice in succession. Then 5 × 10^6^ cells/rat of primary bmMSCs at passage 3 were administered intravenously 7 days after the STZ injection. The untreated T2DM rats were infused with physiological saline. Fasting blood glucose was monitored weekly by Accu-Chek® Performa (Roche Life Science, USA).

### Intraperitoneal glucose tolerance test

The intraperitoneal glucose tolerance tests (IPGTTs) were performed 2, 4, and 8 weeks after the MSC infusion. After overnight fasting, the rats were anesthetized with isoflurane and injected intraperitoneally with 1.5 g/kg glucose. Blood was collected from the tail vein before (0 h) and 0.5, 1, 2, and 3 h after the glucose injection for glucose and insulin testing. Glucose was measured by Accu-Chek® Performa, and serum insulin was measured by radioimmunoassay in the Department of Nuclides, Shandong University.

### Isolation, purification, and protein extraction of islets from rats

Rat islets were isolated from the nondiabetic (NDM) control rats, the nontreated T2DM rats, and the bmMSC-infused T2DM rats (*N* = 3, respectively). The islets were isolated by collagenase digestion (1 mg/ml, type V, catalog no. C9263; Sigma-Aldrich) followed by hand picking under a stereoscopic microscope (Olympus SZX7). In brief, we first separated the common bile duct and ligated it close to the duodenum. A 4.5-sized needle was placed into the engorging bile duct and the backflow of the bile could be observed. We then sacrificed the rat, waited until the duodenum turned pale, clipped the porta hepatis, and injected 6 ml of ice-cold collagenase V (1 mg/ml) slowly into the bile duct to let the pancreas swell. The pancreas was isolated and incubated in 5 ml Hanks solution under 38 °C for 13 min. The pancreas was then shaken with mild wrist force until it disintegrated into a fine sand-like suspension, 20 ml of ice-cold Hanks + 10% bovine serum was added to stop the digestion, and the suspension was passed through a 30-mesh screen. The sample was centrifuged at 1000 rpm for 1 min, then we discarded the supernatant, and washed the sedimentation with ice-cold Hanks solution for three times. To purify the isolated islets, we picked the islets under the stereoscopic microscope. The isolated islets were identified by dithizone (DTZ) staining and about 300 islets could be obtained from a single rat. These islets were lysed in 200 μl RIPA, and the protein extraction procedure was as already described.

### Statistical analysis

Differences between two groups were evaluated using a Student’s *t* test and the χ^2^ test; for three groups or more, a one-way ANOVA was used. *p* < 0.05 was considered statistically significant. All of the statistical analyses were performed with SPSS 16.0 software.

## Results

### Coculturing bmMSCs and MS-1 cells ameliorated H_2_O_2_-induced apoptosis and functional impairment

**Fig. 1:**
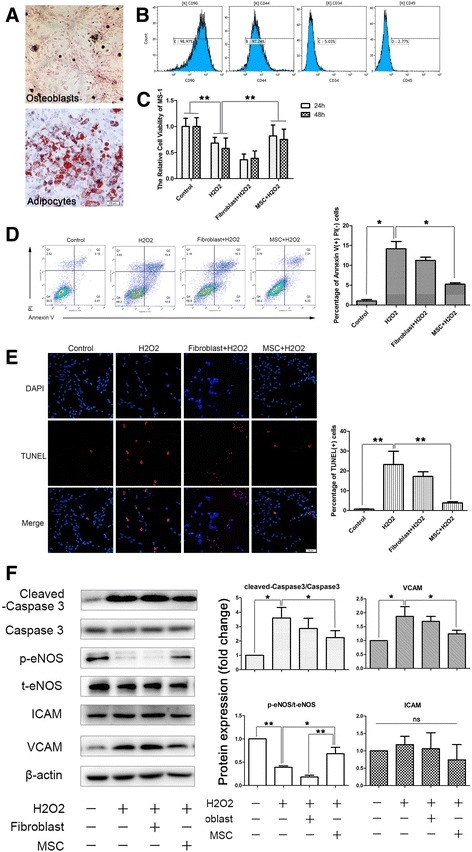
Coculturing bmMSCs and MS-1 cells ameliorated H_2_O_2_-induced apoptosis and functional impairment. **a** MSCs could be induced to differentiate toward osteoblasts (*upper*) and adipocytes (*lower*). Calcium nodes were identified by Van Kossa silver stain (*black*), and lipid droplets were identified by Oil-red O stain (*red*). **b** MSCs were positive for stem cell markers CD90 and CD44 and negative for hematopoietic markers CD34 and CD45, assessed by flow cytometry analysis. **c** MSC-CM reversed the H_2_O_2_-induced cell viability reduction observed by MTT tests. **d, e** Transwell culturing of MSCs with MS-1 ameliorated the H_2_O_2_-induced cell apoptosis, whereas fibroblasts had little influence (**d** shows the results of double staining of annexin V/PI flow cytometry and **e** shows the results of TUNEL staining). **f** Confirmation of the ameliorated cell apoptosis by cleaved caspase 3 western blotting analysis, and the western blotting results of the improved endothelium function indicated by phosphorylated endothelial nitric oxide synthase (*p-eNOS*) and vascular cell adhesion molecule (*VCAM*). Expression of the intercellular cell adhesion molecule (*ICAM*) was not influenced. Quantification of bands performed using Image J software. *p < 0.05, **p < 0.001. *t-eNOS* total endothelial nitric oxide synthase, *MSC* mesenchymal stromal cell, *PI* propidium iodide (Color figure online)

The bone marrow-derived MSCs (bmMSCs) were identified by the expression of multiple surface markers and differentiation toward osteoblasts and adipocytes. After induction, the cells could differentiate toward osteoblasts and adipocytes (Fig. 1a), which were identified by Van Kossa silver stain for the calcium nodes (black) and Oil-red O stain for the lipid droplets (red). The MSCs were positive for stem cell markers CD90 and CD44 and negative for hematopoietic markers CD34 and CD45 (Fig. 1b).

After the identification of MSCs, we then tested the effects of MSCs on oxidative stress-induced endothelium injury. Oxidative stress-induced MS-1 cell injury was established by exogenous administration of 200 μmol/L H_2_O_2_ in cultured MS-1 cells. A significant decline in cell viability was observed by MTT tests (Fig. 1c), and a remarkable elevation in apoptosis was confirmed by annexin V/PI double-staining flow cytometry (Fig. 1d), TUNEL staining (Fig. 1e), and cleaved caspase 3 western blotting (Fig. 1f). Meanwhile, impairment of endothelial function was also observed by the reduction of eNOS phosphorylation and increased expression of adhesion molecule VCAM (Fig. 1f).

However, when MS-1 cells were cultured with MSCs in a transwell coculturing chamber, H_2_O_2_-induced apoptosis declined dramatically, confirmed by both TUNEL staining (Fig. 1e) and annexin V/PI flow cytometry (Fig. 1d). The culture medium (CM) from the MSCs also reversed the H_2_O_2_-induced reduction in cell viability (Fig. 1c) and endothelial nitric oxide synthase (eNOS) phosphorylation, as well as H_2_O_2_-induced caspase3 cleavage/activation and vascular cell adhesion molecule (VCAM) expression, suggesting that MSCs could ameliorate oxidative stress-induced endothelial injury and dysfunction, probably through their paracrine function (Fig. 1f).

### MSCs activated the β-catenin-dependent Wnt signaling pathway in MS-1 cells

**Fig. 2:**
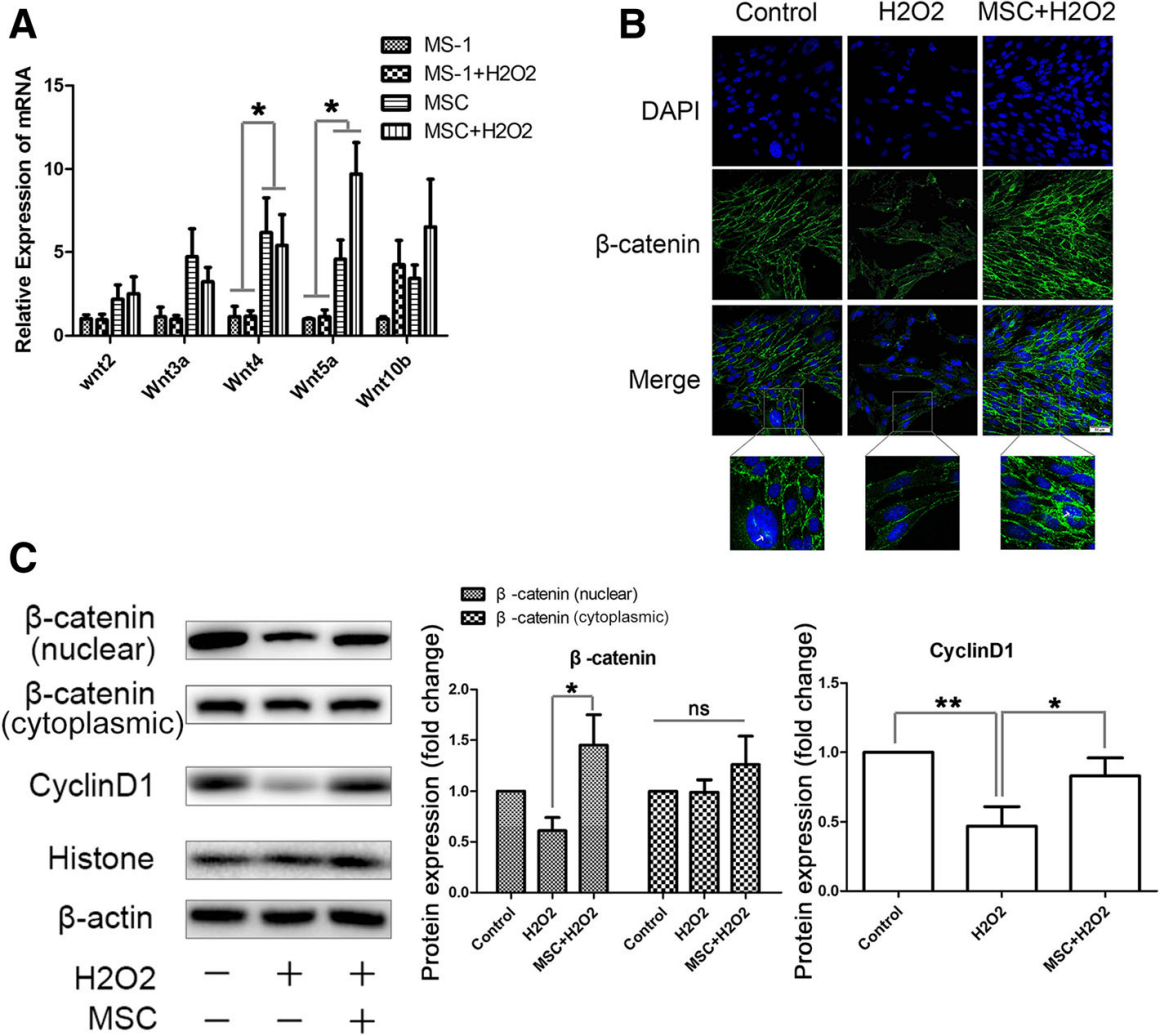
MSCs activated the β-catenin-dependent Wnt signaling pathway in MS-1 cells. **a** Difference in Wnt mRNA expression between the MSCs and MS-1 cells in a transwell coculturing system confirmed by qPCR. **b** Nuclear translocation of β-catenin in the MSC-treated endothelium elevated after coculturing with MSCs, indicated by an increase in FITC-β-catenin fluorescence in the MS-1 nucleus. **c** Confirmation of the increased β-catenin nuclear translocation and the expression of its target gene, cyclin D1, by western blotting analysis. Quantification of bands performed using ImageJ software. *p < 0.05, **p < 0.001. *MSC* mesenchymal stromal cell, *ns* not significant

Wnt proteins are a group of soluble factors that are highly expressed in less mature cells such as stem cells, and their proper functioning is very important for cell self-renewal and stemness maintenance. To explore the possible mechanism for the ameliorative effects of MSCs in oxidative stress-induced endothelial injury, we first analyzed the difference in Wnt mRNA expression between the MSCs and MS-1 cells. We observed a significant increase in the expression of Wnt4 and Wnt5a among all of the Wnts analyzed, including Wnt2, Wnt3a, Wnt4, Wnt5a, and Wnt10b, in the MSCs compared to that of the MS-1 cells, raising the possibility that the Wnt proteins might be involved in the interaction between the two cells (Fig. 2a).

As the Wnt proteins were secreted into the intracellular space, they would bind to the corresponding receptors, such as the Frizzled proteins, and activate the downstream Wnt signaling pathways. To determine whether coculturing with MSCs activated the β**-**catenin-dependent canonical Wnt signaling pathway in MS-1 cells, we analyzed the nuclear translocation of β-catenin in the MSC-treated endothelium. We observed an increase in FITC-β-catenin fluorescence in the MS-1 nucleus after coculturing with MSCs (Fig. 2b). Similar results were shown for the increase in nuclear protein levels of β-catenin in MS-1 cells treated with MSC-CM, whereas the cytoplasmic protein level of β-catenin remained unchanged. We also analyzed the protein expression of cyclin D1, a β-catenin target gene, and found a significant elevation after MSC-CM administration (Fig. 2c). These results suggest that MSCs activated the β-catenin-dependent Wnt signaling pathway in MS-1 cells.

### The beneficial effects of MSCs were partially dependent on the activation of the β-catenin-dependent Wnt signaling pathway

**Fig. 3:**
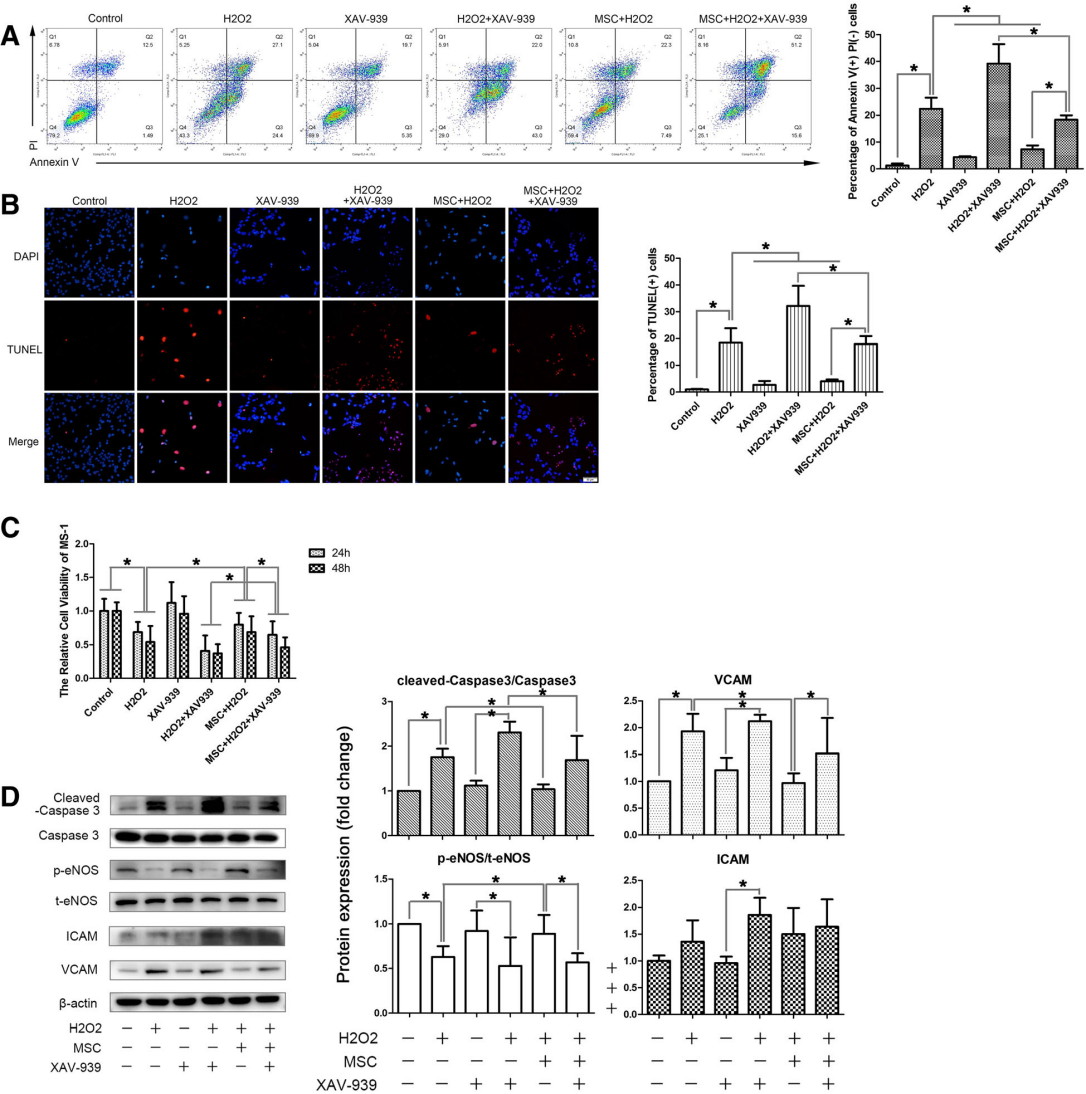
The beneficial effects of MSCs were partially dependent on the activation of the β-catenin-dependent Wnt signaling pathway. **a**, **b** Transwell culturing of MSCs with MS-1 ameliorated the H_2_O_2_-induced cell apoptosis, but the treatment of XAV-939, a Wnt/β-catenin-mediated transcription antagonist, abolished those effects partially (**a** shows the results of double staining of annexin V/PI flow cytometry and **b** shows the results of TUNEL staining). **c** XAV-939 also abolished the MSC-CM-induced cell viability restoration observed by MTT tests. **d** Confirmation of an increase in the apoptosis after XAV-939 treatment by cleaved caspase 3 western blotting analysis, and the western blotting results of the impaired endothelium function indicated by eNOS activation and expression of VCAM. Quantification of bands performed using ImageJ software. *p < 0.05, **p < 0.001. *t-eNOS* total endothelial nitric oxide synthase, *p-eNOS* phosphorylated-endothelial nitric oxide synthase, *ICAM* intercellular cell adhesion molecule, *MSC* mesenchymal stromal cell, *PI* propidium iodide, *VCAM* vascular cell adhesion molecule

To examine whether MSCs act through activating the β-catenin-dependent Wnt signaling pathway in MS-1 cells, we used XAV-939, a Wnt/β-catenin-mediated transcription antagonist that promotes the degradation of β-catenin by stabilizing axin to block the β-catenin-associated effects. XAV itself had little effect on MS-1, but seemed to have a synergistic effect with H_2_O_2_ to induce the apoptosis, eNOS phosphorylation impairment, and VCAM expression upregulation of MS-1. After blocking β-catenin, the protective role of MSCs against H_2_O_2_ was significantly weakened, as shown by a decrease in the cell viability (Fig. 3c), an increase in the apoptosis rate (Fig. 3a, b), impaired eNOS phosphorylation, and increased VCAM expression (Fig. 3d). However, MSCs did actually ameliorate the H_2_O_2_ + XAV-939-induced cell apoptosis to a certain extent, which suggested that the beneficial effects of MSCs were conducted by the activation of the β-catenin-dependent Wnt signaling pathway, at least partially, in the islet microvascular endothelium.

### MSC-secreted Wnt4 and Wnt5a were both involved in the response of MS-1 cells to oxidative stress but may have opposing effects

**Fig. 4:**
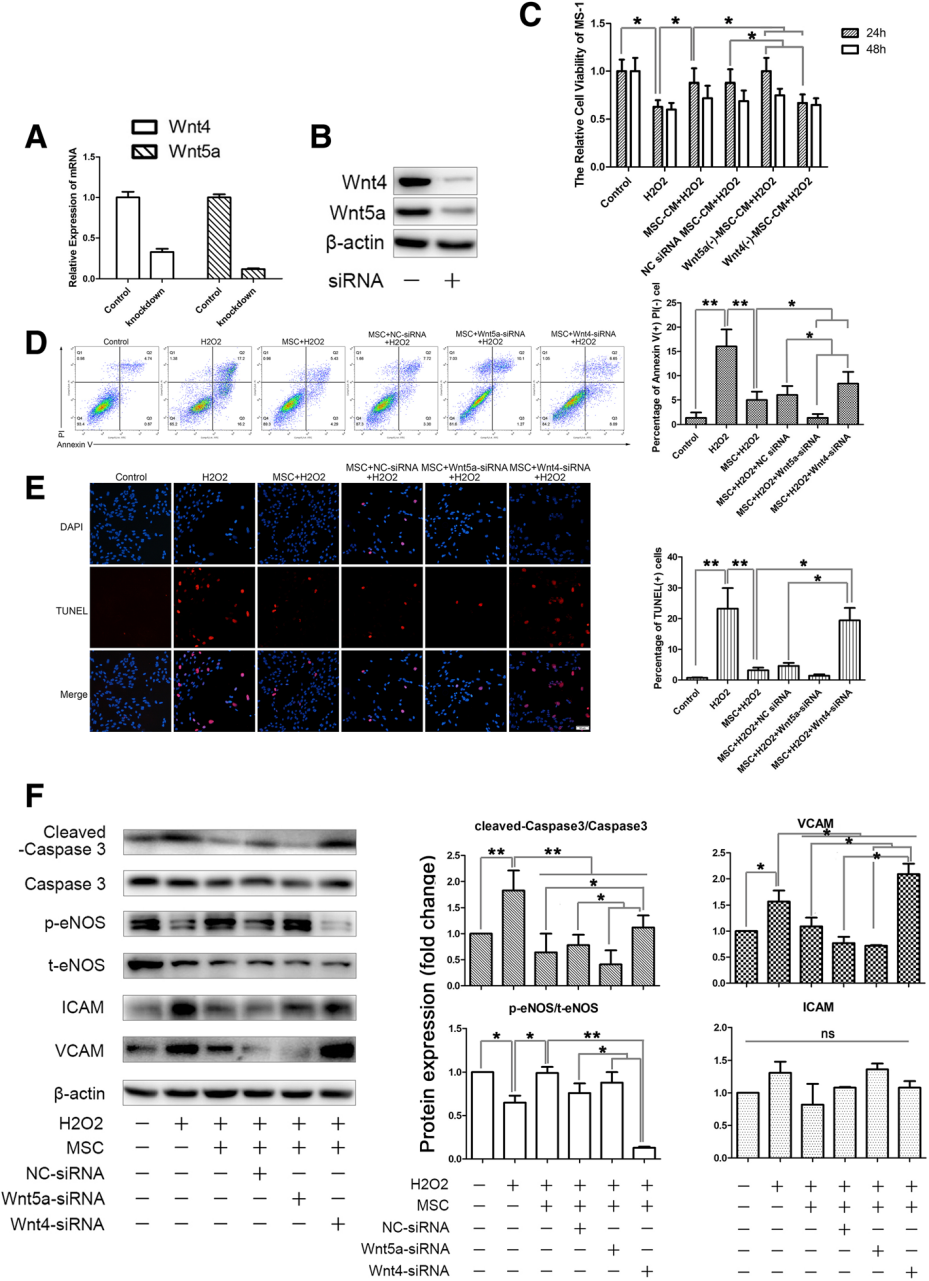
MSC-secreted Wnt4 and Wnt5a were both involved in the response of MS-1 cells to oxidative stress but may have opposing effects. **a**, **b** Confirmation of the knockdown efficiency of Wnt4-siRNA and Wnt5a-siRNA using qPCR and western blotting analysis. **c** MTT cell viability assay suggested that knockdown of Wnt5a further enhanced the beneficial effects of MSC-CM, whereas knockdown of Wnt4 did the opposite. **d**, **e** Transwell culturing of MSCs with MS-1 showed a similar trend; knockdown of Wnt5a in the MSCs improved their anti-apoptosis properties, and knockdown of Wnt4 hampered those effects (**d** shows the results of double staining of annexin V/PI flow cytometry and **e** shows the results of TUNEL staining). **f** Confirmation of changes in the apoptosis of MS-1 after Wnt5a/Wnt4 knockdown in the MSCs by cleaved caspase 3 western blotting analysis, together with the western blotting results of changes in the endothelium function indicated by p-eNOS, ICAM, and VCAM. Quantification of bands performed using ImageJ software. *p < 0.05, **p < 0.001. *CM* conditioned medium, *NC* negative control, *siRNA* silencing RNA, *t-eNOS* total endothelial nitric oxide synthase, *p-eNOS* phosphorylated-endothelial nitric oxide synthase, *ICAM* intercellular cell adhesion molecule, *MSC*, *PI* propidium iodide, *VCAM* vascular cell adhesion molecule

**Fig. 5:**
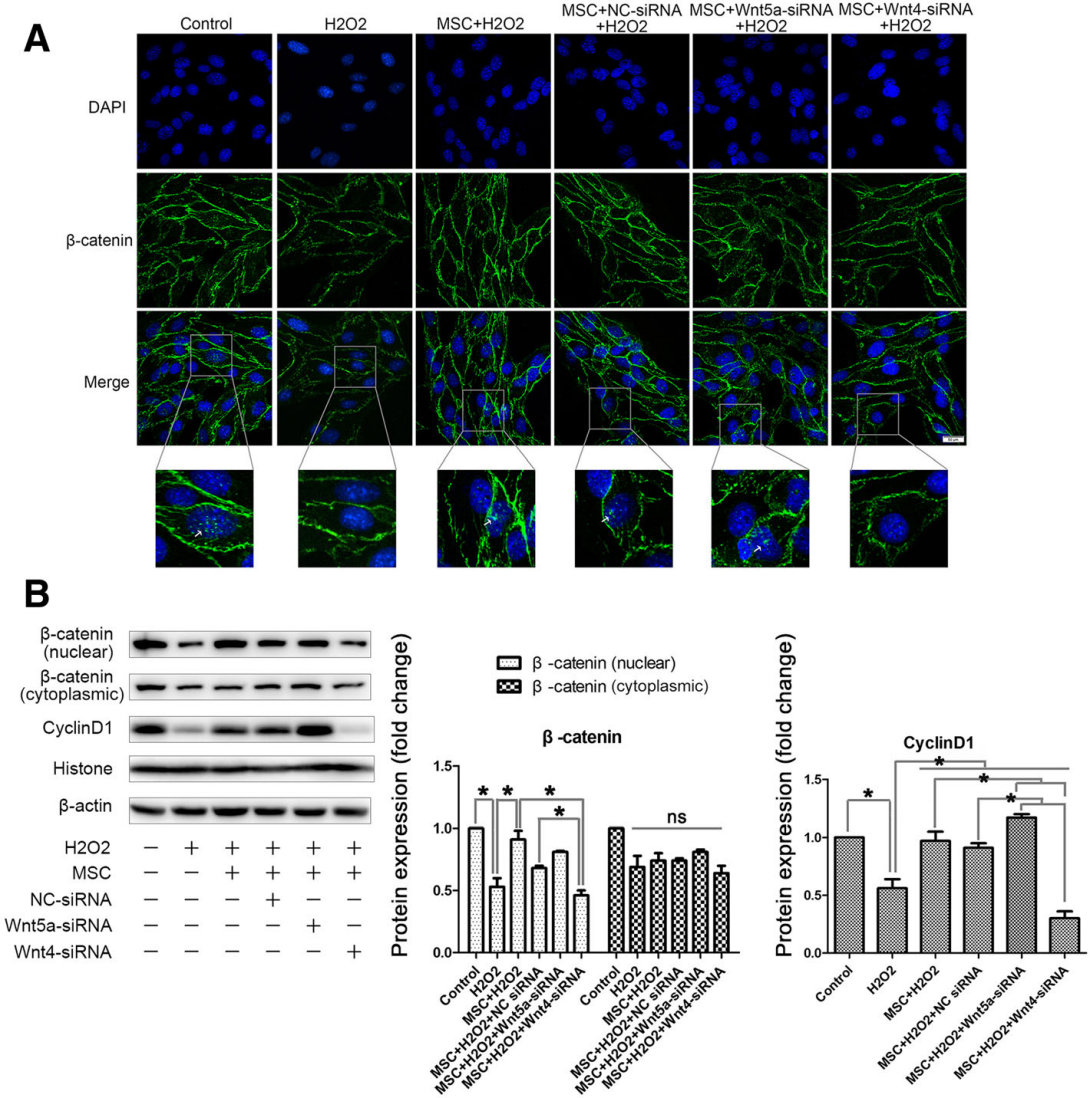
Knockdown of Wnt4 in the MSCs led to a decrease of nuclear translocation of β-catenin in MS-1. **a** Nuclear translocation of β-catenin in the MSC-treated endothelium was abolished by Wnt4 knockdown. However, knockdown of Wnt5a did not result in a significant increase of β-catenin nuclear translocation. **b** Confirmation of the changes of β-catenin nuclear translocation and the expression of its target gene, cyclin D1, by western blotting analysis. Quantification of bands performed using ImageJ software. *p < 0.05, **p < 0.001. *NC* negative control, *siRNA* silencing RNA, *MSC* mesenchymal stromal cell, *ns* not significant

To demonstrate which Wnt protein was involved in the protective process of MSCs toward endothelium injury, we focused on the Wnts, whose expression differs the most between MSCs and MS-1 cells. Therefore, we chose Wnt4 and Wnt5a as possible candidates, and knocked down their expression using siRNAs (Fig. 4a, b). Wnt4 had been proven to activate canonical Wnt signaling pathways in cutaneous cells and artery endothelium, whereas Wnt5a activated the noncanonical Wnt pathways. After knocking down Wnt4 in the MSCs, the ameliorative effects of MSCs were hampered (Fig. 4c–f), which was accompanied by a simultaneous decrease in the nuclear translocation of β-catenin and downstream cyclin D1 expression (Fig. 5). However, Wnt5a knockdown seemed to show the opposite effects: the protective effects were reinforced, and the nuclear protein levels of cyclin D1 were elevated, which suggested that MSC-secreted Wnt4 and Wnt5a both influenced the response of MS-1 cells to oxidative stress but may have opposing effects.

### bmMSC infusion ameliorated hyperglycemia, improved the islet β cell and endothelial function, and increased the β-catenin nuclear translocation in high-fat diet and STZ-induced T2DM rats

**Fig. 6:**
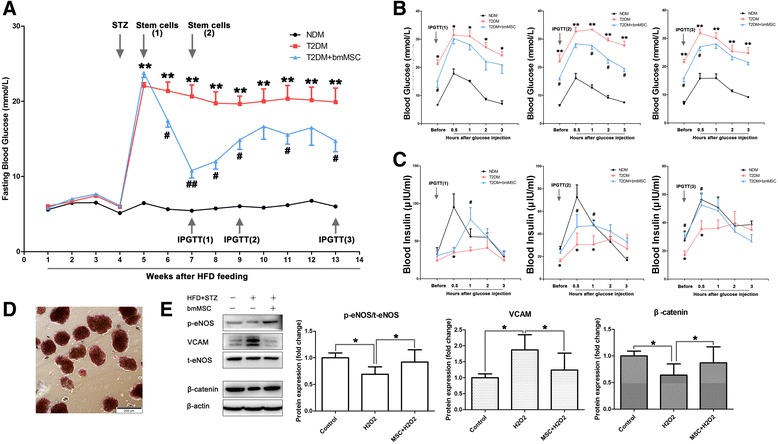
bmMSC infusion ameliorated hyperglycemia, improved the islet β cell and endothelial function, and increased the β-catenin nuclear translocation in HFD and STZ-induced T2DM rats. **a** bmMSC infusion significantly reduced the fasting blood glucose, monitored weekly by Accu-Chek^®^ Performa (Roche Life Science, USA). **b** bmMSCs significantly improved the glucose tolerance during IPGTT 4 weeks after infusion. **c** bmMSC infusion also improved the insulin release during IPGTT 2 and 4 weeks after the infusion, measured by radioimmunoassay. **d** Isolated islets were identified by DTZ staining. **e** Changes of p-eNOS, VCAM, and β-catenin in isolated islets by western blotting analysis. Quantification of bands performed using ImageJ software. Compared with the nondiabetic (*NDM*) control: *p < 0.05, **p < 0.001. Compared with the T2DM group: #*p* < 0.05, ##*p* < 0.001. *bmMSC* bone marrow mesenchymal stromal cell, *HFD* high-fat diet, *IPGTT* intraperitoneal glucose tolerance test, *T2DM* type 2 diabetes mellitus, *t-eNOS* total endothelial nitric oxide synthase, *p-eNOS* phosphorylated-endothelial nitric oxide synthase, *VCAM* vascular cell adhesion molecule

To evaluate the effects of MSCs on islet endothelium in an in-vivo setting, we first built a T2DM rat model by continuous HFD combined with a single dose of STZ (30 mg/kg) at the fourth week of HFD. Then primary rat bmMSCs were administered (5 × 10^6^ cells/rat) 7 days after the STZ injection. We found that the bmMSC infusion significantly reduced the fasting glucose (Fig. 6a), as well as improving the glucose tolerance during IPGTT at the fourth week after MSC infusion (Fig. 6b). The elevated insulin levels during IPGTT at week 4 were also observed (Fig. 6c), so we chose week 4 as our observation point in the following experiments.

Next we managed to evaluate the islet endothelial function by quantifying the expression of p-eNOS and VCAM. In order to rule out the disturbances of the pancreatic endothelium, we isolated the islets from rats 4 weeks after the bmMSC infusion. The isolated islets could be stained red by DTZ (Fig. 6d). Considering eNOS was only expressed in the endothelium, and the only endothelium in the isolated islet should be the islet microvascular endothelium, we took total eNOS (t-eNOS) as the internal reference to measure endothelial p-eNOS and VCAM expression instead of β-actin. The MSC administration significantly elevated the phosphorylation of eNOS, and downregulated the expression of VCAM, thus suggesting that bmMSC infusion could improve the islet microvascular endothelium function in vivo (Fig. 6e).

We also examined activation of the β-catenin-dependent canonical Wnt signaling pathway in the islets. The results of whole islet protein western blotting analysis suggested a significant reduction of β-catenin in the islets of T2DM rats, while MSCs infusion could improve the β-catenin levels (Fig. 6e).

## Discussion

In this study, we investigated the possible mechanisms by which MSCs prevent hydrogen peroxide-induced injury in the MS-1 islet endothelium cell line; by secreting Wnt4 and activating the β-catenin-dependent pathway in MS-1 cells, MSCs could alleviate apoptosis and improve endothelial function by promoting eNOS phosphorylation and reducing the expression of adhesion molecules such as VCAM.

Previous studies have suggested that systemic infusion of MSCs could improve islet function and ameliorate hyperglycemia, but studies focused on islet microcirculation were rare and mostly focused on the impact of cotransplanted MSCs on the islet graft. Rackham et al. [17] reported that cotransplantation of MSCs could increase the endothelial cell number in the graft, and Borg et al.’s [16] study suggested that MSCs hastened the revascularization of the transplanted islet cells, while having no effects on the islet microvascular density. Cao et al.’s [20] work pushed the knowledge of this topic a step further; they suggested that MSCs could enhance the peripheral vascular density of islet grafts by differentiating into vascular smooth muscle cells and endothelial cells and by secreting VEGF. Articles discussing the impact of MSC transfusion on the existing islet microvasculature were even scarcer; Bell et al. [18] transplanted a group of selected MSCs with high-aldehyde dehydrogenase activity into STZ-treated NOD/SCID mice and observed elevated endogenous islet vascular endothelium proliferation. However, due to the technical difficulties of measuring islet blood flow in vivo by microsphere measurements, hydrogen gas clearance, or laser Doppler velocimetry [21], these studies only analyzed the change in endothelium cell numbers but not the function of the islet vasculature or the dilation capacity in particular, which might be the main adaption in hyperglycemia and insulin resistance. Although only an in-vitro study, our study provided evidence that the secreted factors of MSCs could improve the dilation properties of the injured endothelium. By secreting soluble Wnt proteins, MSCs could regulate the activation of the β-catenin-dependent canonical Wnt pathway in MS-1 cells, thus ameliorating oxidative stress-induced cell apoptosis and preventing dilation failure and proinflammatory adhesion molecule upregulation.

The Wnt protein family is a group of soluble proteins that are secreted by less mature cells, such as stem cells and tumors, and together with the activation of Wnt pathways they participate in embryonic development, stemness maintenance, and other pathological processes such as neoplasia formation [22]. Normally, mature cells express low levels of Wnt, but when the cells are under stress Wnt expression might be activated from the former quiescence state to take part in tissue repair [23]. Previous studies have established a link between the Wnt signaling pathway and physiological/pathological angiogenesis [24], but the results seemed to be contradictory, which might be partially due to the different cell types used and the different Wnt signaling pathways activated. Although fewer studies have focused on the relationship between Wnt signaling and islet function, the existing findings appeared to be encouraging. Direct deletion of β-catenin in the maturing beta cells disturbed islet morphology and function, which led to severe deregulation of glucose homeostasis [25]. TCF7L2, a vital participant in the canonical Wnt signaling pathway, has been proven to regulate glucose homeostasis by preserving the beta cell mass [26, 27]. Circulating Wnt proteins have also been shown to be effective in regulating islet function; Wnt3a activated canonical β-catenin-dependent Wnt signaling to promote beta cell proliferation both in vivo and in vitro [28, 29], while Wnt-4 promoted beta cell proliferation but had no obvious impact on secretion [30]. Although there were no articles addressing the effect of Wnt signaling activation on the islet endothelium, it is reasonable to assume that Wnts might also participate in their response to injury.

Previous studies have reported that MSCs could ameliorate tissue injury through secreting various Wnt proteins in dermal cells and other cell types, but we were the first to demonstrate that MSC-secreted Wnt could improve eNOS phosphorylation/activation and reduce VCAM expression in islet endothelial cells after oxidative injury. A study by Zhang et al. [31] demonstrated that MSCs could accelerate the recovery of a cutaneous burn by excreting Wnt4 packaged in exosomes, in which Wnt4 also activated the canonical Wnt pathway and induced the proliferation of dermal cells. In addition, activation of the Wnt pathway by MSCs was also observed in studies by Song et al. [32] and Leroux et al. [33], showing beneficial effects on HCl-induced interstitial cystitis and ischemia-induced muscle fiber injury.

In this study, we also observed an interesting contradiction: MSCs simultaneously secreted “good” Wnts and “bad” Wnts, but the final outcome seemed to be beneficial. The Wnt proteins and the Wnt signaling pathway is a complex network in which different Wnts bind to different receptors, activating antagonistic pathways in a dose-specific and cell-specific manner; the same Wnt protein might activate opposing pathways in different cells or by different concentrations [34]. Take Wnt4 as an example; Wnt4 was discovered as a noncanonical Wnt pathway activator but was later confirmed to be able to act canonically through binding to LRP5 or LRP6 [35]. According to our observation, Wnt4 activated the canonical Wnt pathway in the islet endothelium, which was consistent with what has been reported in dermal cells and muscle fibers [31, 33]. Compared to Wnt4, Wnt5a has been consistently believed to activate a noncanonical Wnt signaling pathway and activates PKC or JNK to conduct a proinflammatory effect [36–38]. In our present study, when MSCs were under oxidative stress, the expression of Wnt5a was significantly elevated, whereas Wnt4 showed a downward trend. Their effects were also observed to be in opposition, in line with previous studies; MSC-secreted Wnt4 was proven to take part in the canonical Wnt signaling activation and partially mediated the protection of MSCs against oxidative stress in islet microvascular endothelium, whereas Wnt5a did the opposite. Fortunately, although MSCs expressed an elevated level of Wnt5a under stress, the anti-inflammation and anti-apoptotic effects were still dominant. It is widely accepted that MSCs could be induced by an inflammatory environment and polarized into two subtypes: a proinflammatory subtype, MSC1; and an anti-inflammatory subtype, MSC2 [39, 40]. However, it is still not clear whether autocrine Wnts could influence the immune polarization of MSCs. Based on this hypothesis, one of our future directions is to determine an intervention to antagonize the proinflammatory environment-induced Wnt5a elevation and Wnt4 downregulation in MSCs in an attempt to minimize the possibility of MSCs polarizing into a proinflammatory subtype and to enhance their beneficial effects.

## Conclusions

In summary, our observations provide evidence that the orchestration of the MSC-secreted Wnts could promote the survival and improve the endothelial function of the injured islet endothelium. Our findings raise the possibility that Wnt4 secreted by MSCs might improve the islet endothelium function by activating the β-catenin-dependent Wnt signaling pathway, but require further in-vivo verification.

## Abbreviations

CM: Conditioned medium
eNOS: Endothelial nitric oxide synthase
FBS: Fetal bovine serum
ICAM: Intercellular cell adhesion molecule
MSC: Mesenchymal stromal cell
NC: Negative control
PBS: Phosphate-buffered saline
RT-qPCR: Reverse transcription quantitative polymerase chain reaction
siRNA: Silencing RNA
T2DM: Type 2 diabetes mellitus
VCAM: Vascular cell adhesion molecule
